# Analysis of the genetic diversity and structure across a wide range of germplasm reveals prominent gene flow in apple at the European level

**DOI:** 10.1186/s12870-016-0818-0

**Published:** 2016-06-08

**Authors:** Jorge Urrestarazu, Caroline Denancé, Elisa Ravon, Arnaud Guyader, Rémi Guisnel, Laurence Feugey, Charles Poncet, Marc Lateur, Patrick Houben, Matthew Ordidge, Felicidad Fernandez-Fernandez, Kate M. Evans, Frantisek Paprstein, Jiri Sedlak, Hilde Nybom, Larisa Garkava-Gustavsson, Carlos Miranda, Jennifer Gassmann, Markus Kellerhals, Ivan Suprun, Anna V. Pikunova, Nina G. Krasova, Elnura Torutaeva, Luca Dondini, Stefano Tartarini, François Laurens, Charles-Eric Durel

**Affiliations:** IRHS, INRA, AGROCAMPUS-Ouest, Université d’Angers, SFR 4207 QUASAV, 42 rue Georges Morel, 49071 Beaucouzé cedex, France; Department of Agricultural Sciences, University of Bologna, Viale Giuseppe Fanin 44, 40127 Bologna, Italy; Plateforme Gentyane, INRA UMR1095 Genetics, Diversity and Ecophysiology of Cereals, 63100 Clermont-Ferrand, France; CRA-W, Centre Wallon de Recherches Agronomiques, Plant Breeding & Biodiversity, Bâtiment Emile Marchal, Rue de Liroux, 4 - 5030 Gembloux, Belgium; School of Agriculture, Policy and Development, University of Reading, Whiteknights, Reading, RG6 6AR UK; NIAB EMR, East Malling Research, East Malling, Kent, ME19 6BJ United Kingdom; Washington State University Tree Fruit Research and Extension Center, 1100 N Western Ave, Wenatchee, WA 98801 USA; RBIPH, Research and Breeding Institute of Pomology Holovousy Ltd., 508 01 Horice, Czech Republic; Department of Plant Breeding, Balsgård, Fjälkestadsvägen 459, Swedish University of Agricultural Sciences, 291 94 Kristianstad, Sweden; Department of Plant Breeding, Swedish University of Agricultural Sciences, Box 101, 230 53 Alnarp, Sweden; Public University of Navarre (UPNA), Campus Arrosadia, 31006 Pamplona, Spain; Agroscope, Institute for Plant Production Sciences IPS, Schloss 1, P.O. Box, 8820, Wädenswil, Switzerland; NCRRIH&V, North Caucasian Regional Research Institute of Horticulture and Viticulture, 39, 40-letiya Pobedy street, Krasnodar, 350901 Russian Federation; VNIISPK, The All Russian Research Institute of Fruit Crop Breeding, 302530, p/o Zhilina, Orel district, Russian Federation; Kyrgyz National Agrarian University, 68 Mederova Street, 720005, Bishkek Kyrgyzstan

**Keywords:** *Malus* x *domestica* Borkh., Genetic resources, Population structure, Variability, SSR markers, Differentiation, Parentage analysis

## Abstract

**Background:**

The amount and structure of genetic diversity in dessert apple germplasm conserved at a European level is mostly unknown, since all diversity studies conducted in Europe until now have been performed on regional or national collections. Here, we applied a common set of 16 SSR markers to genotype more than 2,400 accessions across 14 collections representing three broad European geographic regions (North + East, West and South) with the aim to analyze the extent, distribution and structure of variation in the apple genetic resources in Europe.

**Results:**

A Bayesian model-based clustering approach showed that diversity was organized in three groups, although these were only moderately differentiated (F_ST_ = 0.031). A nested Bayesian clustering approach allowed identification of subgroups which revealed internal patterns of substructure within the groups, allowing a finer delineation of the variation into eight subgroups (F_ST_ = 0.044). The first level of stratification revealed an asymmetric division of the germplasm among the three groups, and a clear association was found with the geographical regions of origin of the cultivars. The substructure revealed clear partitioning of genetic groups among countries, but also interesting associations between subgroups and breeding purposes of recent cultivars or particular usage such as cider production. Additional parentage analyses allowed us to identify both putative parents of more than 40 old and/or local cultivars giving interesting insights in the pedigree of some emblematic cultivars.

**Conclusions:**

The variation found at group and subgroup levels may reflect a combination of historical processes of migration/selection and adaptive factors to diverse agricultural environments that, together with genetic drift, have resulted in extensive genetic variation but limited population structure. The European dessert apple germplasm represents an important source of genetic diversity with a strong historical and patrimonial value. The present work thus constitutes a decisive step in the field of conservation genetics. Moreover, the obtained data can be used for defining a European apple core collection useful for further identification of genomic regions associated with commercially important horticultural traits in apple through genome-wide association studies.

**Electronic supplementary material:**

The online version of this article (doi:10.1186/s12870-016-0818-0) contains supplementary material, which is available to authorized users.

## Background

Cultivated apple (*Malus* x *domestica* Borkh.) is one of the most important fruit crops grown in temperate zones and the most important in the *Rosaceae* family [1]. Although there are more than 10,000 documented apple cultivars worldwide and the apple production area is widespread geographically, the global production is dominated by relatively few cultivars, many of which are closely related [2, 3]. Moreover, in the last century, despite the existence of a large number of apple breeding programs worldwide, only a few well-adapted genotypes (e.g., ‘Red Delicious’, ‘Golden Delicious’, ‘Jonathan’, ‘McIntosh’ or ‘Cox´s Orange Pippin’) were extensively used in apple breeding to release new varieties with desirable traits [2, 4, 5]. The additional release of clonal selections of the most popular and widely grown varieties has further contributed towards the uniformity of commercial apple orchards [6–8]. The gradual replacement of the traditional and locally well-adapted cultivars by a few wide-spread modern varieties has led to a dramatic loss of genetic diversity in the orchards and may also hamper future plant breeding.

The recognition of this situation has encouraged the establishment of action towards the preservation of apple genetic resources worldwide. Multiple apple collections are presently maintained in Europe, preserving mainly old cultivars which have been grown traditionally in their respective regions, but also other cultivars with diverse geographic origins introduced a long time ago, that represent elite selections from before the time of formal breeding. Most of these existing collections were established before molecular identification became available, and in the absence of marker data, the criteria used in the past for selecting the germplasm to be preserved in collections focused mainly on morphology (pomology), eco-geography and/or passport information [9]. The effectiveness of these conservation approaches depends upon the criteria used for selecting germplasm and it has been suggested that genetic diversity may not always be optimal in these, or equivalent collections in other crops [10, 11], and therefore, unintended internal redundancies are expected. Assessment of the genetic diversity in fruit tree species is nowadays mainly performed by marker genotyping techniques [12]. Molecular markers have therefore become an indispensable tool in the management of germplasm collections, and their use is widely applied in characterization to assist and complement phenotypic assessments and to re-examine the composition of the collections [11, 13–16]. The use of molecular markers has not only important implications with regard to the efficiency of the management of the genetic resources, but constitutes a key instrument to evaluate diversity, to elucidate the underlying genetic structure of the germplasm and to quantify relatedness and differentiation between populations among other multiple applications [17–20]. Such knowledge is of high relevance since the conservation of plant genetic resources only fulfills its full potential when they are used effectively, which requires knowledge of the extent and structure of the variation occurring within the material preserved [21].

Until now, the studies of diversity and genetic structure conducted in European apple have been based on the analyses of material from limited geographic areas (mostly nation-scale) [11, 14, 22–26]. By contrast, the extent and structure of the apple genetic diversity conserved at a European level have remained largely unknown. The main obstacle is the different sets of SSR markers used in the different European collections preventing an overall comparison [27]. Thus, in the frame of the EU-FruitBreedomics project [28] a single set of 16 SSR markers was used in a very broad set of apple germplasm (~2440 accessions, mostly of dessert use) preserved in collections located in eleven countries and representing three broad European geographical regions (North + East, West and South) in order to determine the diversity in apple collections at a European scale, to evaluate gene flow in cultivated apple across Europe, as well as to elucidate the stratification of germplasm into population subdivisions and finally, to perform parentage analysis. This is the largest study of apple genetic resources at the pan-European level.

## Results

### SSR polymorphism – identification and redundancy

Among the 2,446 accessions, ten accessions did not show clear PCR amplifications and were discarded from the analysis. Pairwise comparison of multilocus profiles revealed 219 groups of redundancies (Additional file 1), leading to the removal of 405 redundant accessions before further analyses (16 % of redundancy). The number of accessions in each of these identical SSR profile groups varied from two to nine. The cumulative probability of identity (P_*ID*_) was extremely low: P_*ID*_ = 1.3 x 10^−22^, thus highlighting the low risk of erroneous attribution of accessions to duplicate groups. Redundancies were found both within and between collections, leading to the confirmation of numerous previously documented synonyms (e.g., ‘Papirovka’ and ‘White Transparent’, ‘London Pippin’ and ‘Calville du Roi’, or ‘Président van Dievoet’ and ‘Cabarette’) and allowing the putative identification of numerous unknown synonyms or mutant groups (e.g., ‘Gloria Mundi’ = ‘Mela Zamboni’ = ‘Audiena de Oroz’ = ‘Belle Louronnaise’, ‘Court-Pendu Plat/Doux/Gris’ = ‘Krátkostopka královská’, ‘Reinette de Champagne’ = ‘Maestro Sagarra’ or ‘Reinette Simirenko’ = ‘Renetta Walder’ = ‘Burdinche’). Redundancy groups also supported the notion of several national/local name translations such as the English cultivar ‘Cornish Gilliflower’ translated into ‘Cornwallské hřebíčkové’ (i.e., ‘Cornish clove’), or ‘White Transparent’ and ‘Skleněné žluté’ (i.e., ‘yellow glass’) in Czech and ‘Transparente Blanca’ in Spanish, the Russian cultivar ‘Korichnoe polosatoe’ translated into ‘Kaneläpple’ in Swedish (i.e., ‘cinnamon apple’), or the cultivar ‘La Paix’ translated into ‘Matčino’ (i.e., ‘Mother’, a synonym of ‘La Paix’) in Czech. Several cases of homonymy (i.e., accessions with the same name but different SSR profiles) were also found, e.g., three different SSR profiles for the same accession names ‘Pomme Citron’ or ‘Charles Ross’. Data allowed identifying some obvious labeling errors, e.g., X2698 ‘Court Pendu Plat’ which was shown to be the rootstock ‘MM106’, or CRAW-0362 ‘Transparente de Croncels’ which was found likely to actually be ‘Filippa’ (Additional file 1). Following these observations, the apple germplasm dataset was reduced to 2,031 unique genotypes (i.e., exhibiting distinct SSR profiles). Among these individuals, 162 (8 % of the different genotypes) were removed since they had a putative triploid profile, while another ten were discarded because of too much missing SSR data, or because further identified as rootstock or outliers in a preliminary Principal Coordinate Analysis. The final number of unique diploid genotypes further analyzed was therefore 1,859. Using passport data and other accessible information, it was possible to attribute geographical regions of origin (either for three broad designated European regions or, when possible, specific countries) for a large part of the unique genotypes. Roughly 89 % (1,653) of these genotypes could be geographically assigned, with 261, 1,074 and 318 genotypes assigned to Northern + Eastern, Western and Southern historical regions of origin, respectively (Additional file 1). In brief, the Northern + Eastern region was composed of germplasm originating in Nordic European countries plus Russia, the Western region was composed of germplasm originating in Western and Central European countries and the Southern region was composed of germplasm from Spain and Italy (see Methods for more details). The remaining 11 % consisted of either genotypes lacking passport information or genotypes with contradictory information in passport data from different origins. Similarly, the specific country of origin could be attributed to 1,550 genotypes out of the 1,653 geographically assigned (Additional file 1). It is important to note that the European region or country of origin assigned to a genotype was independent from the location of the collection where the sampled accession was maintained, since many collections contained accessions from various origins.

### Genetic diversity across and within European regional groups

The 16 SSR markers amplified a total of 369 alleles across the 1,859 apple accessions used for diversity analysis, ranging from 17 (CH02c09 and CH05f06) to 35 (CH02c06) alleles per locus. The average number of alleles per locus was 23.06, whereas the mean effective number of alleles per locus was 6.59 (Table 1). High average number of alleles per locus and almost identical mean effective number of alleles per locus were noted for the three geographical regions of origin of the germplasm. Allelic richness was normalized to the smallest group (i.e., North + East) to avoid a group size-dependent bias of results. Overall, the results obtained for the material of the three designated regions of origin suggested the existence of a high and relatively homogeneous allelic diversity across Europe (Table 1). Within the 369 alleles identified in the overall set (i.e., across Europe), 73.4 % and 52.0 % were found at frequencies below 5 % and 1 %, respectively (Table 1; data not shown for 1 %). A similar proportion of rare alleles was obtained for the material from the three designated geographical regions of origin, with the exception of alleles detected at a frequency < 1 % with Northern + Eastern and Southern European origins, for which slightly lower percentages were identified (≈38 %). Almost identical mean *He* values were obtained for the overall dataset (0.83) and for the germplasm from each of the three geographical groups (Table 1). Cross-comparison of the allelic composition for the accessions classified into geographic categories showed that 221 out of the 362 alleles (seven alleles appeared only in accessions that could not be classified into geographic groups) were detected in all three geographical groups, 59 alleles (16.3 %) were identified in two geographic groups only, whereas 82 alleles (22.6 %) were specifically found only in one geographic group (i.e., private alleles). At the national level (i.e., countries of origin of the unique genotypes), some countries exhibited a higher rate of private alleles than others: especially, genotypes assigned to Switzerland, Italy and Russia harboured 15, 14 and 14 private alleles (respectively), genotypes from Spain and France harboured 7 private alleles each, whereas genotypes from the Netherlands, Belgium, Great Britain or Sweden had a maximum of one private allele. The pattern of distribution of the frequent alleles (frequency > 0.05) between Southern, Northern + Eastern and Western germplasm was analyzed for each locus separately using Chi^2^ tests. Highly significant differences in the allelic distributions (*P* < 0.001) were found between all the geographic groups for all markers except for the CH-Vf1 locus when comparing Southern and Western germplasm (data not shown).

**Table 1:** Average measures of genetic diversity at two different levels: overall set of accessions and according to the three geographical regions of origin (Northern + Eastern, Southern and Western)

Material	N_A_	N_B_^a^	N_E_	A_R_^b^	Ho	He
Overall set (1859 genotypes)	23.06	16.94	6.59	-	0.81	0.83
European regions of origin						
Northern + Eastern Europe	16.75	10.87	6.24	16.57	0.83	0,82
Southern Europe	17.50	11.87	6.29	16.95	0.81	0.82
Western Europe	20.31	13.94	6.18	16.36	0.81	0.82

^a^Rare alleles were considered if they appeared in a frequency below 5 %

^b^For the geographical European regions of origin, allelic richness was computed after normalization according to the smallest population size (i.e., Northern + Eastern Europe). Number of alleles per locus (N_A_), number of rare alleles (N_B_), effective number of alleles (N_E_), allelic richness (A_R_), and observed (Ho) and expected (He) heterozygosity are included

### Genetic structure and differentiation

**Fig. 1:**
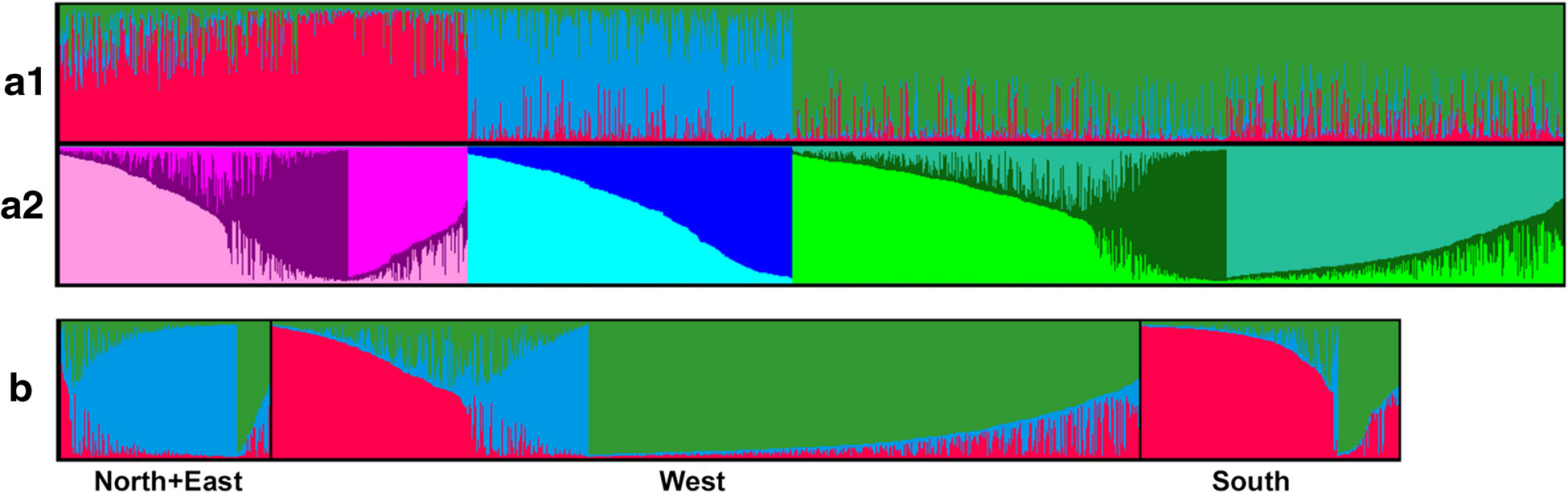
Graphical display of the results of the Structure analyses. **a1**) Proportions of ancestry of 1859 unique diploid apple genotypes for *K* = 3 groups inferred with Structure v.2.3.4 software [67]. Each genotype is represented by a vertical bar partitioned into *K* = 3 segments representing the estimated membership fraction in three groups. The three groups are depicted using the following color codes: Red = group K1; Blue = group K2; Green = group K3. **a2**) Proportions of ancestry of the same 1859 genotypes following a nested Structure analysis within each previously defined group. For K1 and K3 three subgroups are shown and for K2 two subgroups are shown. Each genotype is represented by a vertical bar partitioned into *K* = 2 or 3 subgroups representing the estimated membership fraction in each subgroup. Genotypes are presented in the same order than in a1. The subgroups are depicted using the following color codes: light Pink = K1.1; Purple = K1.2; dark Pink = K1.3; light Blue = K2.1; dark Blue = K2.2; fluorescent Green = K3.1; dark Green = K3.2; light Green = K3.3. **b**) Proportions of ancestry of 1653 unique diploid apple genotypes with known European region of origin for *K* = 3 groups inferred with the same Structure analysis as in a. The genotypes are sorted according to their European region of origin (North + East, West, and South)

**Fig. 2:**
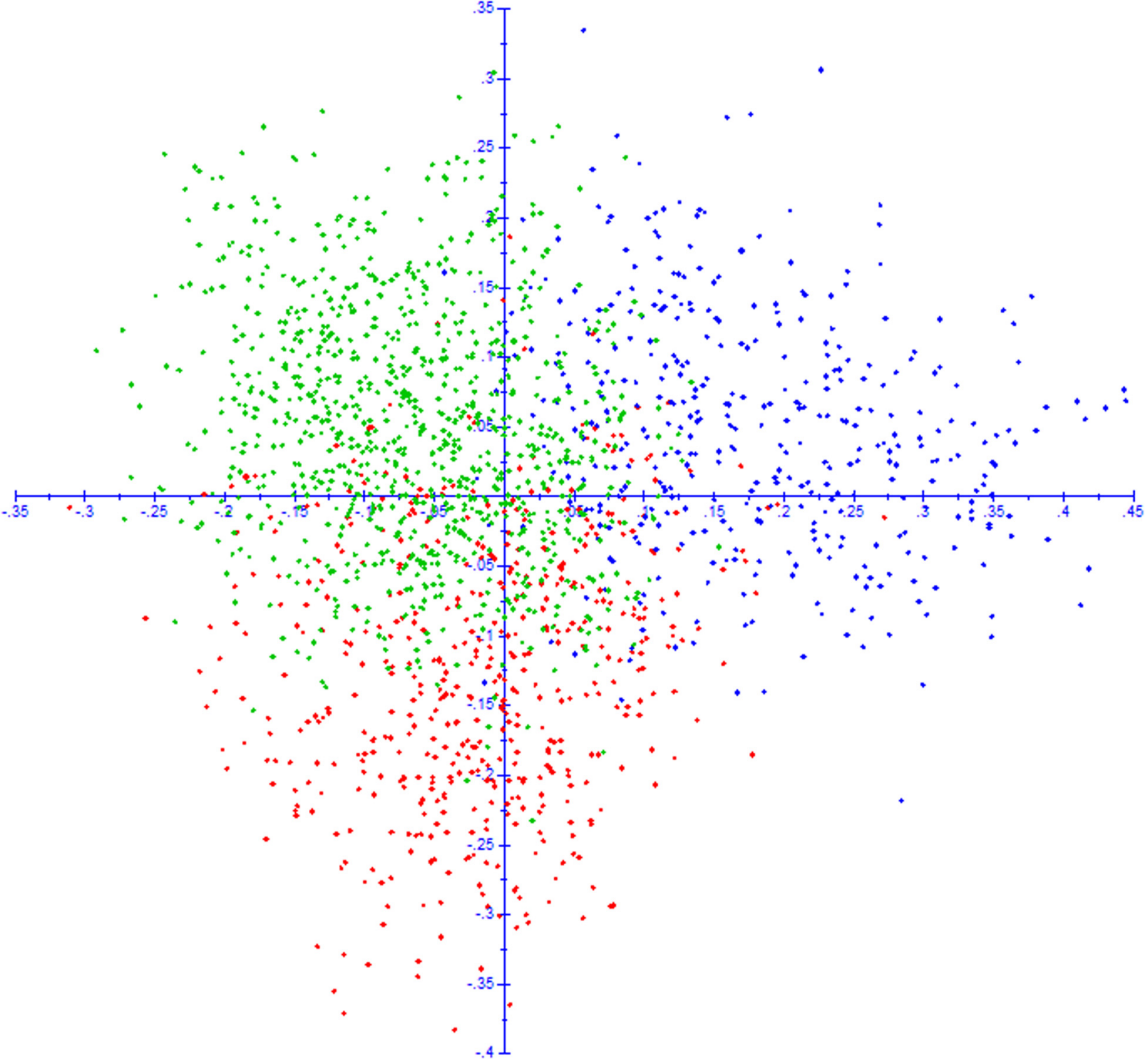
Scatter plot of the Principal Coordinate Analysis (PCoA) of the 1859 apple accessions based on the 16 SSR data. The three groups are depicted using the following color codes: Red = group K1; Blue = group K2; Green = group K3

**Fig. 3:**
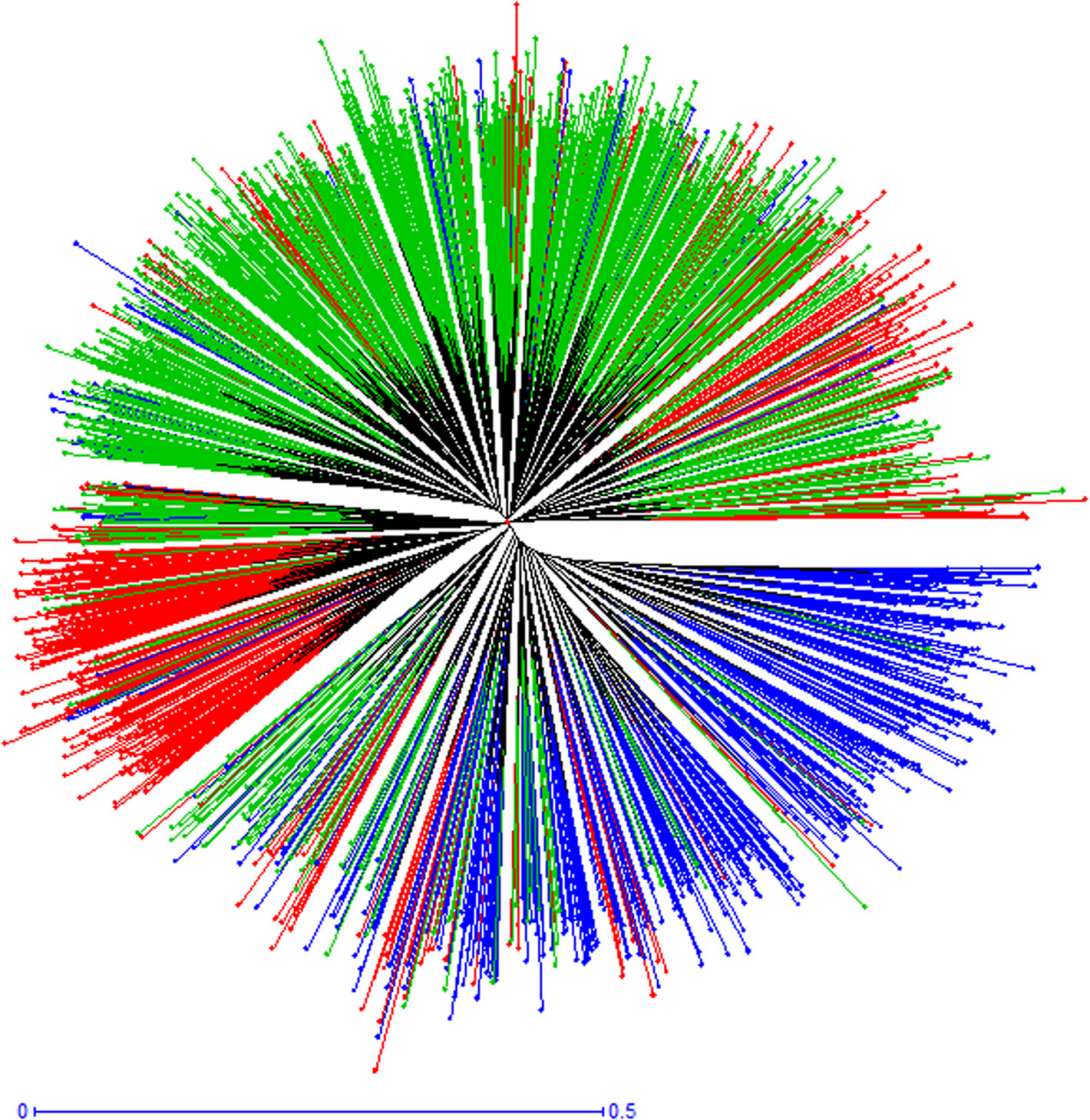
Neighbor-joining dendrogram based on simple matching dissimilarity matrix calculated from the dataset of 16 SSR markers for the 1859 genotypes clustered in the three groups revealed by the Bayesian model-based clustering method. The three groups are depicted using the following color codes: Red = group K1; Blue = group K2; Green = group K3

A Bayesian model-based clustering method was applied to the 1,859 unique diploid genotypes in order to elucidate the underlying genetic structure at a European scale. The analysis of Evanno’s Δ*K* statistic indicated unambiguously *K* = 3 as the most likely level of population stratification (Fig. 1 a1). The mean proportion of ancestry of the genotypes to the inferred groups was 0.81. Using the threshold of *qI* ≥ 0.80 to define strong assignments to groups, 1,175 genotypes (63 %) were identified as strongly associated to a group. This partitioning level corresponded to an asymmetric division of the material into three groups: K1 composed of 506 genotypes, K2 containing 401 genotypes, and K3, the largest group, comprising 952 genotypes. Diversity estimates revealed high levels of allelic variation within each group, with allelic richness ranging between 16.0 (K3) and 18.6 (K1) (Table 2). Genetic discrimination between the three groups was confirmed through a multivariate Principal Coordinate Analysis (PCoA) (Fig. 2). In the bi-dimensional plot, K1 was located mostly to the left of the Y axis, and K2 mostly below the X axis, while K3 occurred to the right of the Y axis and mostly above the X axis. A Neighbor-joining tree also showed three different main clusters (Fig. 3), supporting the identification of the three groups by the Bayesian method.

**Table 2:** Descriptive information for each of the three major groups and eight subgroups of genotypes identified by the Bayesian model-based clustering method

	Number of genotypes in the group/subgroup		He	Number of alleles				Allelic richness
Group/Subgroup	Number Genotypes	Frequency of genotypes with *qI* ≥ 0.8		Total	Private	Unique	A	
K1	506	60 %	0.823	307	34	16	19.19	18.63
K2	401	57 %	0.816	287	23	15	17.94	17.76
K3	952	67 %	0.801	294	22	14	18.36	15.99
K1.1	209	42 %	0.842	282	17	12	17.63	16.38
K1.2	149	54 %	0.789	215	3	1	13.44	13.20
K1.3	148	36 %	0.761	228	6	3	14.25	13.86
K2.1	244	48 %	0.818	268	14	11	16.75	14.73
K2.2	157	53 %	0.778	211	5	4	13.19	12.67
K3.1	375	41 %	0.775	242	7	6	15.13	12.32
K3.2	162	57 %	0.760	171	0	0	10.69	10.31
K3.3	415	51 %	0.809	255	14	8	15.94	13.43

Summary statistics include the partitioning of number of individuals in each group, expected heterozygosity (He), total, private, unique, and average number of alleles (A). Allelic richness is scaled to the smallest group (K2; *N* = 401) or subgroup (K1.3; *N* = 148)

The genetic differentiation between the three designated geographic regions of origin was low (F_ST_ = 0.021, *P* < 0.001, Table 3), suggesting a weak genetic structure for this crop at a European scale in terms of geographical origin. The level of genetic differentiation between the three groups inferred by Structure was only slightly higher (F_ST_ = 0.031, *P* < 0.001). The largest differentiation between pairs of groups was found between Northern + Eastern and Southern germplasm (F_ST_ = 0.042, *P* < 0.001), whereas much lower F_ST_ values were found between the Western and each of the Northern + Eastern (F_ST_ = 0.023, *P* < 0.001) and Southern (F_ST_ = 0.015, *P* < 0.001) materials.

**Table 3:** Analysis of molecular variance (AMOVA) based on the 16 SSR loci of the apple germplasm evaluated in this study corresponding to three regions of origin (Northern + Eastern, Southern and Western Europe) and groups and subgroups defined by Structure analysis

Populations	*df* ^a^		Variance components (%)		
	*W* ^b^	*A* ^c^	*W*	*A*	*p* value
3, geographic origins	1653	2	97.9	2.1	0.001
3, groups defined by Structure	1859	2	96.9	3.1	0.001
3, subgroups of K1	506	2	96.3	3.7	0.001
2, subgroups of K2	401	1	96.6	3.4	0.001
3, subgroups of K3	952	2	97.3	2.7	0.001
8, subgroups (K1+ K2+ K3)	1859	7	95.6	4.4	0.001

^a^ *df* degrees of freedom

^b^ *W* within populations

^c^ *A* among populations

**Fig. 4:**
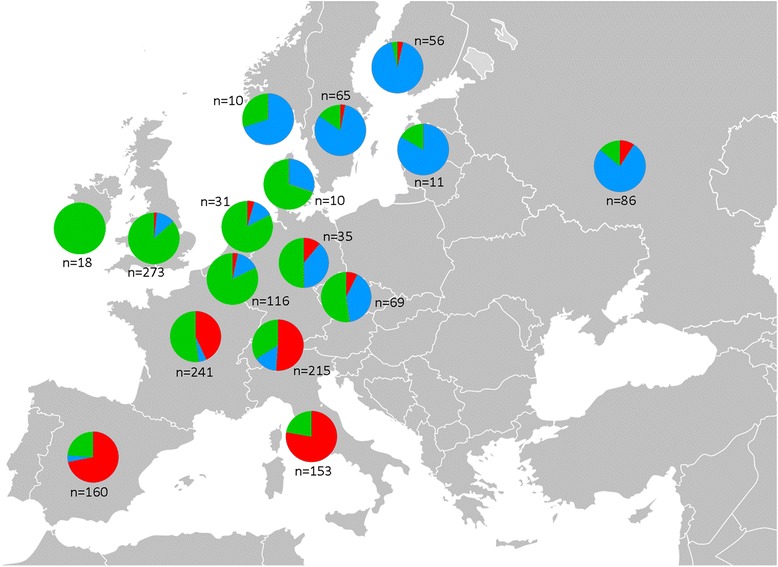
Genetic composition of the groups of cultivars clustered by country of origin for *K* = 3 groups inferred with Structure. For the detailed country list, see Additional file 1. The pies represent the proportion of each group in each country; color codes are as per Fig. 1 a1

The relationship between membership of accessions within the three groups defined by Structure and their geographical regions of origin was also analyzed. 80 % and 75 % of the accessions from Northern + Eastern and Southern Europe clustered in K2 and K1 respectively. The relationship between the material with Western European origin and the third group (K3) was less evident (63 %), but still visible by comparison (Fig. 1b). Although the genetic differentiation revealed between the three groups defined by Structure was not very high, the existence of a relationship between the grouping by geographical regions of origin of the accessions and the three inferred groups is noteworthy. Furthermore, when considering the specific country of origin attributed to the cultivars, the distribution within the three Structure-defined groups appears to follow a clear gradient from North(East) to South of Europe (Fig. 4); the cultivars from Northern Europe and Russia were mainly assigned to the K2 group and the Spanish and Italian cultivars were mainly assigned to the K1 group, with intermediate patterns found for those countries located at the interfaces of the broad regions.

Within the admixed accessions (i.e., *qI* < 0.8) for which the geographical regions of origin (Northern + Eastern, Southern and Western) was known, we defined a membership coefficient threshold (*qI* < 0.55) with the aim of identifying genotypes unambiguously in admixis, in order to examine whether a supplemental relationship could be found between geographical region and grouping by Structure for the admixed material. For the unambiguously admixed material (i.e., *qI* < 0.55) of Southern European origin, the average proportion of ancestry (*qI*) was 0.45 to K1 (the group mostly associated with material from Southern Europe), followed by 0.42 to K3 and 0.13 to K2, the groups mostly composed by material from Western and Northern + Eastern European origins, respectively (data not shown); a slightly less pronounced, but complementary, pattern was observed for the unambiguously admixed germplasm (i.e., *qI* < 0.55) of Northern + Eastern Europe with average proportions of ancestry of 0.43, 0.35 and 0.22 to K2, K3 and K1, respectively. For the unambiguously admixed material (i.e., *qI* < 0.55) of Western origin the average proportion of ancestry to each of these three groups was almost identical (approximately 1/3). This result was in line with the lower F_ST_ values found between the groups K1/K3 (F_ST_ = 0.024, *P* < 0.001) in comparison with the slightly higher differentiation between the groups K1/K2 (F_ST_ = 0.039, *P* < 0.001) and K2/K3 (F_ST_ = 0.036, *P* < 0.001). The dispersion of the three groups in the PCoA plot was also in agreement with these results, showing the highest overlap between K1 and K3 followed by K2 and K3.

### Nested-Bayesian clustering approach: substructuring of the diversity

In order to investigate the substructuring of the diversity within each of the three groups identified in the initial analysis we used a nested application of the Structure software. To do this, the three groups were analyzed independently. To evaluate the strength of the hypothetical subdivisions (i.e., subgroups) within each group, simulations for each *K* value were examined, paying attention to the internal consistency between the runs, the mean proportion of ancestry of accessions within each subgroup, and the proportion of accessions unequivocally assigned (*qI* ≥ 0.80).

The analysis of the relationships between *K* and Δ*K* for K1 suggested a probable subdivision of this material into three subgroups and the assignment of genotypes was well correlated between runs. The average proportion of ancestry for the accessions clustered in the three subgroups of K1 was 0.75, with 44 % of the accessions showing strong assignments. Two subgroups for K2 and three for K3 were similarly established. In both cases, the assignment of genotypes was well correlated between runs, and almost identical average proportions of ancestry to those for the subgroups of K1 were obtained with slightly higher proportions of strongly assigned accessions (47 % and 50 % respectively). Secondary peaks at other *K* values were also explored but these subdivisions had less statistical support (data not shown). Therefore, we adopted eight subgroups as the most suitable partitioning degree of substructuring (Fig. 1 a2). For these eight subgroups the affinity of almost half of the individuals (47 %) to their respective subgroups was strong and the assignment of admixed accessions was consistent between runs. The examination of the eight subgroups showed considerable differences in size, ranging from 148 (K1.3) to 415 (K3.3) genotypes, and variable proportion of accessions strongly assigned to the inferred subgroups (Table 2). K3.2 was the subgroup with the highest proportion of strongly assigned genotypes (57 %), whereas K1.3 had the highest proportion of admixed accessions. The proportion of accessions unambiguously assigned for the remaining six subgroups ranged from 41 % to 54 %, whereas the mean proportion of ancestry for the accessions clustered in each one of the eight subgroups was very stable (≈0.75).

The analysis of the relationship between the different subgroups and the putative countries of origin of the germplasm indicated potentially interesting correlations, especially for groups K1 and K3. About 70 % of the subgroup K1.2 consisted of germplasm originating from Spain. Similarly, 46 % of the subgroup K1.1 and 50 % of the subgroup K1.3 consisted of germplasm originating from Switzerland and Italy, respectively (Additional file 2); the latter subgroup was also composed of a further 39 % of the cultivars with a French origin and interestingly, a significant proportion of these were attributed to Southeastern France (data not shown). The disentangling of the substructuring pattern therefore allowed not only the dissection of the internal distribution of the diversity within group K1, but also the detection of three subgroups strongly associated with some particular countries of origin. With respect to the collections from the Northern + Eastern part of Europe (Sweden, Finland and Russia), no clear differentiation of the germplasm in the two subgroups of K2 was observed (Additional file 2). For the subgroup K3.1, about half of the germplasm consisted of cultivars from either the United Kingdom or France. All of the 40 cultivars selected in the French collection as being recently bred, clustered in a single small subgroup (K3.2) which was mostly composed of English, US and, perhaps more surprisingly, Spanish cultivars. Major standard cultivars such as ‘Golden Delicious’, ‘Red Delicious’, ‘Jonathan’ and ‘Ingrid Marie’ were also assigned to this subgroup, as well as ‘Cox’s Orange Pippin’ and ‘James Grieve’. Interestingly, most of the 40 cider apple cultivars (87 %) were assigned to one subgroup (K3.3) which was mostly composed of French, English, and Swiss cultivars. The other standard cultivars were assigned to the latter subgroup and to subgroup K3.1.

Genetic diversity estimates were calculated for all the subgroups obtained by the nested Bayesian model-based clustering (Table 2). While *He* ranged from 0.76 (K1.3 and K3.2) to 0.84 (K1.1), indicating a high level of heterozygosity contained in all the subgroups, the percentage of alleles represented in each one of the eight subgroups was very variable, ranging from 46 % (K3.2) to 76 % (K3.1). Some private alleles were identified in all subgroups except for K3.2. They were most abundant in K1.1, but a considerable number of them were found also in K3.3 and K2.1. Most of the private alleles (approx. 72 %) were also unique as they were identified in only one accession. To properly evaluate the allelic diversity between the eight subgroups, we applied a rarefaction approach to compensate for the differences in subgroup size. The allelic richness obtained for the eight subgroups supported the previous results, confirming the highest diversity in K1.1 and the lowest diversity in K3.2.

Estimates of genetic differentiation showed that only 3.7 % (K1) and 3.4 % (K2) accounted for variation among subgroups within groups (Table 3). The genetic differentiation between the subgroups into which K3 was subdivided was considerably lower (Table 3). Considering the eight subgroups obtained by the overall Nested Bayesian approach, the results showed that variation among subgroups accounted for 4.4 % of the total variation. Regarding the F_ST_ pairwise tests between subgroups (Table 4), irrespective of whether they belonged to the same group or not, the highest F_ST_ corresponded to the pair K1.3/K2.2 (F_ST_ = 0.087, *P* < 0.001), followed by K1.3/K2.1 (F_ST_ = 0.077, *P* < 0.001), and the lowest to the pairs K1.1/K3.3 (F_ST_ = 0.016, *P* < 0.001) and K3.1/K3.3 (F_ST_ = 0.023, *P* < 0.001).

**Table 4:** Pairwise estimates of F_ST_ among the eight subgroups obtained by the nested Bayesian clustering approach

Subgroup	K1.1	K1.2	K1.3	K2.1	K2.2	K3.1	K3.2	K3.3
K1.1	—							
K1.2	0.030	—						
K1.3	0.035	0.051	—					
K2.1	0.028	0.067	0.077	—				
K2.2	0.049	0.076	0.087	0.035	—			
K3.1	0.034	0.051	0.061	0.061	0.055	—		
K3.2	0.051	0.065	0.070	0.070	0.058	0.029	—	
K3.3	0.016	0.042	0.060	0.038	0.051	0.023	0.038	—

All the estimates were highly significant (*P* < 0.001)

### Parentage reconstruction

Two-parents-offspring relationships within the 1,859 diploid genotypes were explored using CERVUS software. A total of 46 putative trios (offspring and two inferred parents) were identified with high (95 %) confidence level. These consisted of two already documented trios, (‘Calville Rouge du Mont Dore’ and ‘Belle de Mleiev’ and their parents; [23]), as well as another 10 recent and 34 old cultivars (Table 5). The two parents of the 10 modern cultivars, for which full parentage was already documented were correctly inferred (e.g., ‘Heta’, ‘Jaspi’ and ‘Pirkko’ = ‘Lobo’ x ‘Huvitus’, ‘Pirja’ = ‘Huvitus’ x ‘Melba’, or ‘Mio’ = ‘Worcester Pearmain’ x ‘Oranie’). In most cases, the two parents of the older cultivars were not known and thus newly inferred (Table 5). Inferred parentage was found for old cultivars from various European countries (6x for Italy; 4x for Great Britain, Switzerland, Czech Republic, and Sweden; 3x for Germany and Spain; 2x for Belgium). Perhaps unsurprisingly, some accessions were more frequently inferred as parents, such as the two French cultivars ‘Reine des Reinettes’ (= ‘King of the Pippins’) or ‘Transparente de Croncels’ which were each identified three times. Geographic convergence of parentage was frequently observed (e.g., ‘Kramforsäpple’ = ‘Sävstaholm’ x ‘Åkerö’, all three from Sweden; ‘Beauty of Moray’ = ‘Keswick Codlin’ x ‘Stirling Castle’, all three from Great Britain; ‘Roja de Guipuzcoa’ = ‘Urte Sagarra’ x ‘Maxel Gorri’, all three from Spain; or ‘Scodellino’ = ‘Abbondanza’ x ‘Decio’, all three from Italy). But hybridizations between cultivars from distant countries were also observed (e.g., ‘Rotwiler’ presumably from Switzerland = ‘King of the Pippins’ x ‘Alexander’ from France and Ukraine, respectively; or ‘Godelieve Hegmans’ from Belgium = ‘Red Astrakan’ x ‘Transparente de Croncels’ from Russia and France, respectively). It should be noted that the female and male status of the inferred parents could not be specified from the available SSR markers.

**Table 5:** Full parentages of 46 apple cultivars inferred within the set of the 1859 apple unique accessions using 16 SSR markers with their accession codes, accession names (AcceNumber), their duplicate codes according to the SSR profile (FBUNQ) and their putative country of origin (OriginHist)

Offspring ID	Accename	FBUNQ	OriginHist	First candidate ID	Accename	FBUNQ	OriginHist	Second candidate ID	Accename	FBUNQ	OriginHist	Status ^j^
X1618	Calville Rouge du Mont Dore	963	FRA	BAL086	Alexander	30	UKR	DCA_I05	Mele Ubriache^a^	361	FRA	doc.
X1846	Belle de Mleiev	1563	-	X0557	Mc Intosh	508	CAN	1957218	King of the Pippins	37	FRA	doc.
BAL035	Heta	1774	FIN	CRAW-0433	Lobo	788	CAN	FIN09	Huvitus	4922	FIN	recent
BAL039	Jaspi	1776	FIN	CRAW-0433	Lobo	788	CAN	FIN09	Huvitus	4922	FIN	recent
FIN18	Pirkko	4930	FIN	CRAW-0433	Lobo	788	CAN	FIN09	Huvitus	4922	FIN	recent
BAL010	Rödluvan	107	SWE	CRAW-0433	Lobo	788	CAN	BAL023	Barchatnoje	1768	RUS	recent
BAL109	Arona	1819	LVA	CRAW-0433	Lobo	788	CAN	BAL112	Iedzenu	1822	LVA	recent
BAL176	Nyckelby	1861	SWE?	CRAW-0433	Lobo	788	CAN	1957188	Cox's Pomona	2033	GBR	recent?
BAL059	Pirja	444	FIN	FIN09	Huvitus	4922	FIN	CRAW-0836	Melba	167	CAN	recent
FIN43	Pirkkala	4949	FIN	BAL042	Kaneläpple	512	RUS	FIN14	Lavia	4926	FIN	recent
BAL154	Mio	543	SWE	CZ_G2D_0045	Worcester parména	550	GBR	BAL056	Oranie	48	SWE	recent
BAL052	Oberle	1784	CAN	BAL027	Early Red Bird	236	CAN	CRAW-0266	Stark Earliest	468	USA	old
BAL091	Förlovningsäpple	1804	SWE	CHE0893	Heuapfel	1248	CHE	X1646	Saint Germain	31	-	old
BAL167	Valldaäpple	1853	SWE	CHE0893	Heuapfel	1248	CHE	BAL179	Göteborgs Flickäpple	1863	SWE	old
BAL099	Kramforsäpple	1811	SWE	BAL161	Sävstaholm	573	SWE	BAL195	Åkerö	308	SWE	old
BAL158	Stenkyrke	463	SWE	BAL171	Fullerö	1857	SWE	CZ_LJ_0045	Malinové podzimní^b^	722	POL	old
FIN07	Finne	4920	FIN	BAL161	Sävstaholm	573	SWE	FIN08	Grenman	4921	FIN	old
1942035	Beauty of Moray	1925	GBR	2000053	Keswick Codlin	1438	GBR	2000090	Stirling Castle	2103	GBR	old
1951242	Brighton	2011	NZL?	X4915	Red Dougherty	939	NZL	CZ_LC_0411	Hlaváčkovo^c^	23	USA	old
1957208	Ben's Red	2035	GBR	CRAW-0020	Devonshire Quarrenden	622	GBR	1955077	Box Apple	2025	GBR	old
1965004	Fred Webb	2054	GBR	1946088	Winter Marigold	324	GBR	1957181	Gascoyne's Scarlet	45	GBR	old
2000083	Rivers' Early Peach	2099	GBR	2000051	Irish Peach	2093	IRL	BAL169	Aspa	1855	SWE	old
BMN0011	Roja de Guipuzcoa	3854	ESP	BMN0017	Urte Sagarra	956	ESP	BMN0171	Maxel Gorri	3896	ESP	old
BMZ016	Cella	3935	ESP	BMN0022	Erreka Sagarra	957	ESP	X5102	Bisquet	535	FRA	old
BMN0070	Madotz-01	3869	ESP	1957218	King of the Pippins	37	FRA	X7201	Transparente de Croncels	62	FRA	old
CHE1322	Rotwiler	1271	CHE?	1957218	King of the Pippins	37	FRA	BAL086	Alexander	30	UKR	old
CHE1788	Roseneggler	3718	CHE	1957218	King of the Pippins	37	FRA	CZ_BoN_0429	Trat. Laze	2284	CZE	old
CHE0032	Ernst Bosch	1003	DEU	1947074	Ananas Reinette	69	NLD	CZ_GF_0415	Evino^d^	7	GBR	old
CHE0168	Eibner	3258	CHE	CRAW-0836	Melba	167	CAN	CZ_BoN_0424	Trevínské červené^e^	71	USA	old
CHE1390	Klefeler	3589	CHE	KRAS123	Papirovka	25	RUS	X7199	Rose de Berne	83	CHE	old
CRAW-0226	Laubain n°1	2126	BEL	CRAW-0086	Bismarck	3	AUS	CZ_GS_0478	Ušlechtilé žluté^f^	90	GBR	old
CRAW-0105	Godelieve Hegmans	2116	BEL	BAL175	Röd Astrakan	82	RUS	X7201	Transparente de Croncels	62	FRA	old
CZ_BB_0442	Nathusiovo	2268	DEU	CZ_GL_0464	Bláhovo Libovické	2311	CZE	X7201	Transparente de Croncels	62	FRA	old
CZ_BB_0434	Panenské veliké	2265	CZE	CZ_GP_0469	Panenské české	1529	CZE	X1344	Reinette de Landsberg	61	DEU	old
CZ_GK_0412	Proche	2308	CZE	CRAW-0425	Calville Rouge d'Automne	13	FRA	X1344	Reinette de Landsberg	61	DEU	old
CZ_BoN_0421	Moravcovo	2283	CZE	CZ_GP_0469	Panenské české	1529	CZE	CHE0269	Pomme Bölleöpfel	1377	-	old
CZ_GL_0456	Bláhův poklad	694	-	CZ_GL_0464	Bláhovo Libovické	2311	CZE	CZ_GG_0438	Malinové hornokrajské^g^	47	NLD	old
CZ_GP_0473	Petr Broich	2321	DEU	1957175	Annie Elizabeth	15	GBR	2000075	Peasgood's Nonsuch	51	GBR	old
CZ_BB_0458	Šarlatová parména	2269	CZE	CZ_GG_0442	Malinové holovouské	452	CZE	X8233	Petite Madeleine	24	-	old
CZ_BB_0466	Podzvičinské^h^	231	-	X0691	Boiken	108	DEU	X1071	Reinette de Caux	629	NLD	old
DCA_017	S.Giuseppe	1646	ITA	DCA_090	Abbondanza	327	ITA	DCA_C44	Rambour Frank (MI)	493	FRA	old
DCA_H03	Scodellino	1642	ITA	DCA_090	Abbondanza	327	ITA	DCA_E52	Decio	397	ITA	old
DCA_E72	Gelato Cola	330	ITA	DCA_E69	Gelato (CT)	780	-	DCA_F74	Limoncella (TN)^i^	708	ITA	old
DCA_H62	Liscio di Cumiana	1713	ITA	DCA_H29	Carla	114	-	DCA_C21	Renetta di Grenoble	263	ITA	old
DCA_I96	Ros Magior	1658	ITA	DCA_I80	Rus d' Muslot	321	-	X1115	Rome Beauty	334	USA	old
DCA_F47	Mela Golden Simile di Villa Collemandina	1692	ITA	DCA_A20	Rosa Mantovana (TN)	101	ITA	CRAW-0025	Yellow Bellflower	77	USA	old

^a^DCA_I05 'Mele Ubriache' duplicate with 'Calville Rouge d'Hiver' [23]

^b^based on 11 SSR [64] the accession CZ_LJ_0045 'Malinové podzimní' was shown to be duplicated with 'Danziger Kantapfel'

^c^based on 11 SSR [64] the accession CZ_LC_0411 'Hlaváčkovo' duplicate with 'Northen Spy'

^d^based on 11 SSR [64] the accession CZ_GF_0415 'Evino' duplicate with 'Mank's Codlin'

^e^based on 11 SSR [64] the accession CZ_BoN_0424 'Trevínské červené' duplicate with 'King David'

^f^ based on 11 SSR [64] the accession CZ_GS_0478 'Ušlechtilé žluté' duplicate with 'Golden Noble'

^g^based on 11 SSR [64] the accession CZ_GG_0438 'Malinové hornokrajské' duplicate with 'Framboise'

^h^based on 11 SSR [64] and on 13 SSR [14] the accession CZ_BB_0466 'Podzvičinské' duplicate with 'Altlander Pfannkuchenapfel' and 'Thurgauer Kent'

^i^based on 11 SSR [64] the accession DCA_F74 'Limoncella' (TN) duplicate with 'Cola'

^j^recent or old cultivars ; doc. = inferred parentage already documented in [23]

## Discussion

### Identification and redundancy

The exchange of genotyping data between research units has increased considerably in recent years, with the aim to investigate the extent and distribution of diversity for specific crops at a wide geographic scale. In this study, the application of a common set of 16 SSR markers on a wide set of dessert apple cultivars distributed across three broad European regions allowed the detection of redundant accessions and duplicated genotypes between and within collections, and the description of the structuration of a significant part of the European apple diversity. Cross-comparison of SSR data in attempts to combine datasets from multiple sources has often been problematic due to challenges in harmonizing the allelic sizes between different laboratories [18, 29, 30]. By combining existing data over numerous shared reference accessions in our collections with the re-genotyping of a subset of the accessions, we were able to strongly secure the SSR allele adjustment over sites. This dataset represents a highly valuable resource for the comparison of apple germplasm collections throughout Europe and the rest of the world. Taking into consideration the rich allelic diversity present in the European apple germplasm, it would be useful to identify a relatively small set of varieties that offer a good representation of the allelic variability identified in this germplasm to act as an internal control (i.e., a reference set) between laboratories for future use.

Interestingly, duplicate groups involving accessions from different collections underlined some putative drift in the cultivar denomination. Some good examples were ‘Pott’s seedling’ and ‘Pottovo’ (FBUNQ14), or ‘Signe Tillish’ and ‘Signatillis’ (FBUNQ34). In addition, ‘sports’ are often given derivative names (e.g., ‘Crimson Peasgood’ as a sport of ‘Peasgood’s Nonsuch’) but the current analysis was not set up to distinguish between clones and ‘sports’ of cultivars with potential morphological differences. Many likely errors in denomination of genotypes were also detected when multiple representatives of a given cultivar were detected within a group, but a single supposed representative was obviously outside of the group and was often associated with representatives of a different cultivar. For example, ‘Drap d’Or’ and ‘Chailleux’ (FBUNQ92) are known to be synonyms used in France for the same cultivar, and accession DCA_D35 ‘Drap Dore’, which was found to belong to the group FBUNQ50, was most likely a denomination error since almost all other members of this group were ‘Winter Banana’. In other cases, accessions with uncertain denomination could be resolved, such as CRAW-1858 ‘Reinette Baumann?’ (FBUNQ21) and accession CRAW-1108 ‘Peasgood Nonsuch?’ (FBUNQ51) for which the molecular analyses confirmed that they were most likely ‘true-to-type’ cultivars. The question of ‘trueness-to-type’ is a major issue in apple germplasm management where extensive budwood exchange between regions and countries has occurred for centuries. Indeed, an erroneously denominated accession can be transmitted from collection to collection for years, such that a large number of representatives within a duplicate group (as per the present study) should not always be considered definitive proof of the trueness-to-type of accessions but this objective evidence is extremely valuable in highlighting issues to resolve. Since genebank curators have often collected material of old cultivars from private gardens or from tree pasture orchards, unidentified or misidentified material can later be detected either by classical phenotypic characters and/or by using genetic markers. As an example, this study showed that an old so called local cultivar ‘Madame Colard’ (CRAW-0365 – FBUNQ72), described to have been raised in 1910 by the nurseryman Joseph Colart at Bastogne (Belgium), exhibited the same SSR profile as the old English apple cultivar called ‘Royal Jubilee’ (UK-NFC 2000085) raised already in 1888. Further comparison with historical descriptions could conclude that they are the same cultivar. Additional insights from the passport data of accessions would be needed to help in tracing the transmission of the material from collection to collection and pomological characterization will be required to compare accessions to published descriptions of the variety. This will remain a task for the curators of collections, in order to improve curation of germplasm in a coordinated way.

It is important to note that the criteria used to select the accessions at the country-level were not always the same. For instance, the INRA and UNIBO material corresponded to former “core collections” built to encompass a large variability not restricted to the national/local accessions [23, 24]. Conversely, the UK-NFC and FRUCTUS material was restricted to older diploid accessions considered to derive from UK and Switzerland, respectively. A similar, despite less stringent situation was applied also for CRA-W, RBIPH, SLU, and the Spanish accessions (UPNA, UDL and EEAD). For MTT, NCRRI, VNIISPK, and KNAU, the national representativeness was more limited and strictly restricted to accessions considered to be emblematic landrace cultivars. The germplasm was thus somewhat heterogeneous in nature, but still allowed a broad examination of the European dessert apple diversity. In the future, it will be useful to enlarge the dataset to include additional accessions from the collections considered here as well as other European collections [11, 31] or collections from other regions worldwide [32–34] to provide a wider perspective on genetic resource conservation of apple worldwide.

### Genetic diversity

The high level of diversity and heterozygosity in apple germplasm at a European level agreed with previous results obtained at collection-scale in several European countries, e.g., Italy [24], Spain [26], France [23], Sweden [22], Czech Republic [25] or Switzerland [14]. The large diversity found is consistent with the weak bottleneck effect reported in connection with the domestication of this species [35–37]. Probably a combination of factors are involved: i) vegetative propagation methods that have been adopted since ancient times favoring the dispersal of cultivars across geographic regions [38, 39], ii) forced allogamy due to the self-incompatibility system of *Malus* × *domestica* [40], iii) multiple hybridization events at each geographical region combined with human activities, e.g., selection and breeding [36, 37] and, iv) diversifying selection associated with adaptive criteria for the subsistence in diverse agricultural environments [41, 42]. Interestingly, the distribution of private SSR alleles over the countries of origin of the unique genotypes was somewhat unbalanced at the European level with much higher occurrences in genotypes assigned to Switzerland, Italy or Russia than in genotypes originating from Northern-Western Europe. Whilst these findings should be considered with caution because of possible biases linked to the initial sampling or to the size differences of the genotype sets, this study underlines that accessions originating from Southern Europe and Russia could be expected to bring original genetic diversity into modern breeding programs especially for traits related to more extreme climate adaptation. Overall, the highly diverse germplasm studied here contains much more genetic variation than do modern apple cultivars, many of which having been selected for optimal performance within a narrow range of environmental conditions [5, 37, 42].

### Coordinated actions: a key point for better knowledge of the resources conserved

This large-scale analysis in apple germplasm constitutes a good example of the efficiency and value of coordinated international actions to enhance the knowledge of diversity conserved at a European level. The results obtained offer a valuable step to undertake actions to coordinate European resources towards optimizing the management of apple germplasm across Europe in line with the aspirations of the European Cooperative program on Plant Genetic Resources (ECPGR). The results also offer a potential starting point that may open new opportunities for apple breeding in the near future. All breeding advances are built upon the diversity available, and a key role of the germplasm collections is to help safeguard natural forms of genetic variation and to make them accessible to plant biologists, breeders, and other key users [15]. The extensive germplasm evaluated in this study consisted mainly of old and/or locally grown accessions across Europe, many of which remain underutilized in cultivation or breeding programs. The preservation of traditional cultivars in living germplasm collections must be regarded as an invaluable reservoir of insufficiently explored genetic diversity that may become useful for apple breeding in a near future, and the establishment of coordinated genetic data is hoped to increase the accessibility of this material to breeding programs. From the perspective of modern-day fruit production, most of these old varieties would now be considered as obsolete since they are not particularly well-adapted to current agricultural practices and marketing. Nevertheless, this material should be considered as a reservoir of potentially interesting genes to be used for further improvement. This is particularly relevant in a crop like apple, for which the current production is highly dependent on a very limited number of cultivars with a narrow genetic basis for the bulk of current production [5]. As an example, it can be mentioned that 50 % of the commercially marketed apple production in the European Union consists of only four cultivars, ‘Golden Delicious’, ‘Gala’, ‘Idared’ and ‘Red Delicious’ [43]. The low diversity of the subset of elite cultivars used for commercial production during recent decades is likely to result in a bottleneck hampering future genetic improvement [37]. The recognition of this situation should encourage the establishment of coordinated actions across different levels (regional, national and international scales) to define strategies for the efficient conservation of the genetic resources of this species.

### Genetic structure: major divisions and substructuring of the diversity

The attribution of country of origin to traditional cultivars can be a matter of endless debate, especially for those dating back two-three centuries or more. Initial descriptions in pomologies and booklets can be subject to errors in denomination confused by historical distribution and renaming, resulting in synonymy, as well as the re-use of old names for more recent findings or misidentifications. This is less problematic for the better known old cultivars as many of them have been widely documented and monitored over years in several countries. However, for local cultivars and/or landraces where less information is available, the correct attribution can be complicated, especially between neighboring countries. It is also worthy of note that the ‘country of origin’ relies on a political construct, which can be prone to significant change within the potential lifetime of many varieties of apple (and other long lived perennial crops). Therefore, we first used a conservative approach and discussed our findings in terms of three broad European regions of origin. Then, we analyzed the structuration at a country-scale, but noting that the exact attribution of a given country to a genotype was not always unanimously agreed so that this finer level of analysis should be considered with an element of caution.

Using a Bayesian model-based clustering method we were able to initially discern the existence of three robust groups reflecting major divisions of the germplasm. These groups were linked with the three geographical regions of origin, although differentiated only to a low degree. This would reflect a situation whereby the cultivars from a given region were more frequently derived from crosses between parental cultivars from the same region than from cultivars from elsewhere. Nevertheless, the migration of the plant material associated to human movement together with hundreds of years of empirical selection may have caused a significant gene flow across Europe. This is clearly indicated by the low genetic differentiation between groups and has shaped the overall pattern of genetic diversity. A spatially and temporally dynamic process where seeds and mainly graftwood were exchanged between geographically distinct populations has contributed to the increase of the genetic diversity in each area through unintentional gene flow or human-mediated intentional crosses [35, 36, 44]. The background common to other long lived tree fruits, including factors such as multiple origins of cultivated populations, ongoing crop-wild species gene flow and clonal distribution of genotypes together with the features associated with fruit tree species (lengthy juvenile phase, extensive outcrossing, widespread hybridization or mechanisms to avoid selfing) has defined the way they evolve in nature and resulted in extensive population genetic variation, but limited population structure [44]. A possible cause of divergence between the three identified groups could be the differential adaptation to distinct environmental conditions as are the case between Southern, Western and Northern + Eastern Europe. A similar situation was postulated for grapevine cultivars where the genetic structure appeared to be strongly shaped by geographic origin and intentional selection [13]. But since selection causes differentiation in particular regions of the genome on which selection pressure is acting [45], another likely cause of the population structure is genetic drift (i.e., changes in allelic frequencies caused by chance events) as also shown in e.g., apricot [46]. Together with selection, migration and drift can shape the local adaptation of species [47].

Although there may have been some mistakes in attributing cultivars to country of origin, the genetic makeup of the cultivars at the European level clearly appeared to show a North-East to South gradient. Interestingly, some countries exhibit intermediate marker data patterns in consistency with their intermediate geographic positions. This was clearly manifested at the national scale for the German and Czech cultivars which were shared between K2 and K3 groups. Similarly, the French and Swiss cultivars were shared between K1 and K3 groups. By contrast, cultivars from Southern Europe (Spain and Italy), from Northwestern Europe (United Kingdom and Ireland, Belgium, the Netherlands), and from North + Eastern Europe (Sweden and Finland) and Russia were mostly assigned to a single group (K1, K3, and K2, respectively). For the admixed germplasm from Southern and Northern + Eastern European geographical regions of origin, a certain degree of introgression with the Western germplasm was also indicated in contrast to the low contribution of the Northern + Eastern germplasm into the Southern germplasm and viceversa. Thus, in agreement with the correspondence between clustering and regions or countries of origin of the germplasm, the geographical proximity appears to align with the patterning observed in the admixed accessions.

In cases demonstrating the presence of a significant hierarchical population structure as this study suggests, this method preferentially detects the uppermost level of structure [26, 48–50]. As a consequence, when large datasets in species with a complex background are analyzed, it is possible for an underlying substructure to remain undetected within the major divisions of the germplasm. In this context, the “*nested (or two-steps) Structure*” clustering method has been shown to be an efficient tool to delineate further levels of substructure in both apple and other plant species [10, 24, 26, 49–52]. In this study, the three groups inferred from the first round of Structure analysis were used as the starting point for revealing internal substructuring. Eight subgroups were identified with remarkable differences in both allelic composition and richness, as well as a considerable number of private alleles associated to particular subgroups. Nevertheless, the relationship between the placement of the genotypes in the subgroups and their country of origin varied considerably between subgroups in contraposition to the clearer and more consistent clustering trend within the three groups. As discussed earlier, this stratification may reflect historical processes of selection and adaptation to local conditions that might suggest a “*fine-delineation*” of the intra-variation within each main geographical region of origin. This is most probably the case for the K1.1 subgroup which mainly consists of Spanish cultivars and could reflect a process of both local adaption and isolation by distance related to the Pyrenean barrier. For the K1.3 subgroup, local adaptation to the Southern region could be inferred together with a potential for more intense commercial exchange between Italy and Southern France. For other subgroups, the relationship with particular countries or small regions was not obvious, but some interesting associations between subgroups of group K3 and recent cultivars and some of their founders or particular usage (cider apple cultivars) could be noticed.

### Relatedness and family relationships

The previously reported parentage of 10 recent cultivars was correctly inferred in all cases. These results served as a control and validated the parentage assignment obtained with the CERVUS software [53] indicating that the number and informativeness of SSR markers were sufficient at least for these cultivars. The 16 SSR markers were nevertheless limited in their ability to infer parentages, and additional cases might have been detected with a larger number of SSR markers. In a recent paper [54], it was suggested that the number of 27 SSR loci used in that study was a minimum to be utilized for full parentage reconstruction. Basically, the LOD score tests used in the CERVUS software are computed according to the SSR allelic frequencies, and thus, parentages involving common alleles are more difficult to detect. By contrast, parentages involving low frequency and rare alleles are more easily detected. On that basis, it is worthy to note that the more frequently detected parents (i.e., ‘Reine des Reinettes’ = ‘King of the Pippins’, and ‘Transparente de Croncels’) are possibly representing a biased view of the frequently involved parents, as they most probably carry rare or low frequency alleles in at least some SSR loci. Putative parents present in the dataset but carrying more common alleles may have been hidden because of the statistical limits of their detection with 16 SSR markers. A similar situation was observed by [23] with the frequent appearance of ‘Reine des Reinettes’ as a parent of four old cultivars out of 28, using 21 SSR markers. In the near future, medium and high density SNP arrays [55–57] will provide much more power to infer parentages.

The parentage of some old cultivars was either confirmed, in the case of ‘Ernst Bosch’ = ‘Ananas Reinette’ x ‘Mank's Codlin’ (synonym: ‘Evino’) or augmented, in the case of ‘Ben’s Red’ = ‘Devonshire Quarrenden’ x ‘Box Apple’ (Table 5) where the second parent was initially hypothesized to be ‘Farleigh Pippin’ [58]. Distances between the geographic origins of the inferred parents (when known), ranged from crosses between geographically close cultivars to crosses between very distant cultivars, reflecting the large gene flow across Europe caused by, e.g., extensive exchange of budwood over centuries.

Some traditional folklore about the origination of old apple cultivars could be either substantiated or refuted by the SSR-based parentage information. As one example, the old Swedish cultivar ‘Förlovningsäpple’ is said to derive from a locally acquired seed in Northern Sweden where only a few cold-hardy apples can be grown. The two unknown parents were here inferred to be the Swiss cultivar ‘Heuapfel’ and the wide-spread cultivar ‘Saint Germain’ (X1646) also known as ‘Vitgylling’ in Sweden, a name used for a group of more or less similar, white-fruited, early-ripening and winter-hardy cultivars. Interestingly, the ‘Vitgylling’ accession included in the present study (BAL072) did not have the same SSR profile as ‘Saint Germain’, but they share one allele for all 16 SSR loci and may therefore be related. In two other cases, traditional Swedish folklore indicated that a sailor brought an exotic seed to the island of Gotland and to Kramfors in Northern Sweden, respectively, resulting in ‘Stenkyrke’ and ‘Kramforsäpple’. For ‘Stenkyrke’, one parent is the Swedish ‘Fullerö’ and the second is the German cultivar ‘Danziger Kantapfel’ which has been much grown in Sweden. The origin of ‘Stenkyrke’ is thus probably much more local than anticipated. Similarly, the surmised American sailor origin of the seed giving rise to ‘Kramforsäpple’ is refuted by the fact that the parents of this cultivar are the Swedish ‘Sävstaholm’ and ‘Åkerö’.

It is important to keep in mind that trueness-to-type of the accessions is not guaranteed, thus the labeling of the offspring or the parents can be erroneous in some cases. Conversely, the inferred parentages are robustly established so that the genetic relationships between the accessions are valid independently of their names. Crosses between the two inferred parents could be performed to reproduce the cross which gave birth to the offspring cultivar, especially if genetic analysis of some particular traits of the latter genotype indicates an interesting application in plant breeding.

## Conclusions

The analysis of a large and representative set of *Malus* x *domestica* genotypes indicated that apple germplasm diversity reflects its origination within three main geographic regions of Europe, and that a weak genetic structure exists at the European level. This structuring of genetic variation in European dessert apple is caused by evolutionary processes relevant to the domestication of perennial fruit species with factors such as gene flow created by, e.g., ancient roads of commerce across the continent, other human activities like intentional selection and later breeding, and genetic drift. The remarkable differences in the allelic variation found at group and subgroup levels of germplasm stratification constitute a strong indication of that the diversity is hierarchically organized into three *genepools*, with consistent evidence of a pattern of internal substructure. The potential value for modern fruit production is mostly unknown since a majority of the accessions are poorly evaluated from an agronomic point of view. Thus, phenotypic data obtained with standardized methods is required to determine the commercial potential of the preserved material and to enable its use in new crosses to increase the genetic basis of the cultivated apple.

The integration of data for collections from different European geographic regions using standardized methods will undoubtedly form an important step in developing the European strategy for conservation of apple germplasm and constitute the starting point to define a European “apple core collection”. This will constitute a decisive step in the field of conservation genetics, and may also have direct implications on the improvement of our understanding of the species, including i) the identification of genomic regions associated with commercially important horticultural traits, ii) the discovery of new germplasm features that may be taken advantage of for efficient breeding and iii) the analysis of genotype x environmental interactions for studying the stability of the most economically important traits for this species.

## Methods

### Plant material

Apple germplasm collections from nine European countries, plus Western part of Russia and Kyrgyzstan, were available for this study (Additional file 1): France (INRA, Institut National de la Recherche Agronomique, 399 accessions), Italy (UNIBO, University of Bologna, 216 acc.), Belgium (CRA-W, Centre Wallon de Recherche Agronomique, 408 acc.), Czech Republic (RBIPH, Research and Breeding Institute of Pomology Holovousy, 263 acc.), United Kingdom (UK-NFC, University of Reading, 310 acc.), Sweden (SLU, Swedish University of Agricultural Sciences, 199 acc.), Finland (MTT Agrifood Research, 50 acc.), Spain (UPNA, Public University of Navarre, UDL, University of Lleida, and EEAD, Aula Dei Experimental Station, 269 acc.), Switzerland (FRUCTUS, Agroscope, 237 acc.), Russia (NCRRIHV, North Caucasian Regional Research Institute of Horticulture and Viticulture, and VNIISPK, The All Russian Research Institute of Horticultural Breeding, 83 acc.), and Kyrgyzstan (KNAU, Kyrgyz National Agrarian University, 12 acc.). In all countries, the accessions were mostly chosen as old local/national dessert cultivars (registered or at least known before 1950), but 12 standard dessert cultivars were also included to strengthen comparisons between collections, namely ‘Golden Delicious’, ‘Red Delicious’, ‘McIntosh’, ‘Rome Beauty’, ‘Granny Smith’, ‘Jonathan’, ‘Winter Banana’, ‘Ingrid Marie’, ‘Ananas Reinette’, ‘Reinette de Champagne’, ‘Discovery’ and ‘Alkmene’. Moreover, 40 old cider apple cultivars and 40 recently-bred dessert cultivars were sampled in the INRA collection in order to investigate particular patterns. Altogether, 2,446 accessions were thus considered (Additional file 1). Available collections were somewhat heterogeneous in nature as some of them corresponded to already established core collections (INRA and UNIBO) whereas others were selected for the present study thanks to available SSR marker data (UK-NFC and FRUCTUS, see below), or were chosen as a subset of mainly local cultivars (CRA-W, RBIPH, SLU, MTT, UPNA, UDL, EEAD, NCRRIHV, VNIISPK and KNAU). Cultivars that were known to be triploid or duplicated were avoided since this analysis was performed with an aim to subsequently use a major part of the material in a Genome Wide Association Study to be carried out within the EU FruitBreedomics project [28].

### SSR genotyping

A set of 16 SSR markers developed by different groups [59–62] was used to genotype the 2,446 accessions (Additional file 3). These SSR markers are distributed over 15 out of the 17 apple linkage groups, and 15 of them are included in a former list recommended by the ECPGR *Malus/Pyrus* working group [63]. The 16^th^ marker of this list, NZ05g08, was replaced by the marker CH-Vf1 because the former showed either complex scoring pattern or low level of polymorphism in previous studies [23, 26]. SSR marker data were fully available for the collection from INRA [23]. SSR data were available (i.e., for some, but not all of the 16 SSR markers) for collections from UK-NFC [64], FRUCTUS [14], UPNA, UDL and EEAD [26], and UNIBO [24], so that only the missing SSR marker data were generated in the present study. Fully new SSR datasets were generated for collections from CRA-W, RBIPH, SLU, MTT, NCRRIHV, VNIISPK, and KNAU.

Forward primers were labeled with four different fluorescent dyes (6-FAM, VIC, NED, or PET) in order to be combined into four different multiplexed reactions (Additional file 3). Polymerase chain reactions (PCR) for the four multiplex PCRs were performed in a final volume of 11 μL using 10 ng of DNA template, 0.18 μM of each primer (with the exception of some markers as described in Additional file 3), and 1× PCR Master mix of QIAGEN kit multiplex PCR (Qiagen, Hilden, Germany). PCR cycling conditions were as follows: pre-incubation for 15 min at 94°C, followed by 4 cycles using a touchdown amplification program with an annealing temperature reduced by 1°C per cycle from 60°C to 55°C, followed by 34 cycles, each consisting of 30 s denaturing at 94°C, 90 s annealing at 55°C, and 60 s elongation at 72°C, the last cycle ending with a final 15-min extension at 72°C. SSR amplification products were analyzed with an ABI3730 XL sequencing system (Applied Biosystems, Foster City, CA, USA). Fragment analysis and sizing were carried out using GeneMapper v.4.0 software (Applied Biosystems, Foster City, CA, USA); chromatograms were independently read by two operators. When SSR marker data were already available and obtained at different sites, SSR allele sizes were carefully adjusted between collections, both by use of reference accessions known to be in common between collections and by re-genotyping a subset of each collection with the full set of 16 SSR markers to confirm the allele adjustment.

### Diversity assessments

The multilocus SSR profiles were compared pairwise in order to establish the genetic uniqueness of each accession. Accessions were considered as duplicates if they had identical SSR fingerprints, or if they had one allelic difference for a maximum of two SSR loci thus making room for some genotyping errors and/or spontaneous SSR mutations. On this basis, redundant profiles were removed from the dataset to avoid bias in genetic analyses and duplicate groups were labeled with unique group ID codes (FBUNQ codes). An accession was declared as a putative triploid when at least three of the 16 SSR loci exhibited three distinct alleles. Analyses of descriptive diversity statistics were conducted at locus level. For each SSR marker, SPAGeDi v.1.3 software [65] was used to estimate the number of alleles (*N*_*A*_), the number of alleles with a frequency below 5 % (*N*_*B*_), the number of effective alleles *(N*_*E*_), and the observed (*Ho*) and expected (*He*) heterozygosity. The probability of identity (P_*ID*_) was calculated as follows [66]:

$$ {P}_{ID}={\displaystyle \sum {p}_i^4}+{\displaystyle \sum {\displaystyle \sum \Big(2{p}_i}}{p}_j\Big){}^2 $$

where *pi* and *pj* are the frequencies of the i^th^ and j^th^ alleles and i ≠ j. The cumulative P_*ID*_ over the 16 SSR was computed as the product of the P_*ID*_ of each individual marker.

### Determination of the geographical regions of origin of the unique genotypes

Using passport data along with reviewing published records with a focus on old literature (national compilations/varietal catalogues/reports) and specialized websites we were able to discern the geographical regions of origin for a large part of the unique genotypes analyzed. This was further helped by the resolution of identified duplicates and comparison of accessions against additional SSR data of the whole UK-NFC apple collection kindly made available from the UK-NFC database [64] and of the whole FRUCTUS collection kindly made available by Agroscope [14]. We first decided to define three broad historical European regions of origin of the germplasm according to geographical proximity and traditional agricultural relations between them: North + East (Sweden, Norway, Finland, Denmark, Baltic countries, plus Russia, Ukraine and Kyrgyzstan), West (Ireland, United Kingdom, France, Belgium, the Netherlands, Switzerland, Germany, Czech Republic) and South (Spain and Italy). When available, countries of origin of the cultivars were also documented although, this information should be considered with caution since the information on the countries of origin was not always fully consistent within duplicates groups.

### Analysis of the genetic structure

The software Structure v.2.3.4 [67] was used to estimate the number of hypothetical subpopulations (*K*) and to quantify the proportion of ancestry of each genotype to the inferred subpopulations. No prior information about the geographical origin of the accessions was considered in the analysis. Ten independent runs were carried out for *K* values ranging from two to 10 using 500,000 Markov Chain Monte Carlo (MCMC) iterations after a burn-in of 200,000 steps assuming an admixture model and allelic frequencies correlated. In order to assess the best *K* value supported for our dataset, the Δ*K* method [68] was used through the Structure harvester v.0.6.93 website [69] to examine the rate of change in successive posterior probabilities over the range of *K* values. When the results described above suggested additional substructuring of the diversity in subgroups, a second-level (nested) application of the Structure clustering method was carried out analyzing separately each of the *K* major groups previously obtained [10, 24, 26, 50, 51]. Genotypes were assigned to the group (or subgroup) for which they showed the highest membership coefficient, considering an accession strongly assigned to each partitioning level if its proportion of ancestry (*qI*) was ≥0.80 [70–72]; otherwise they were considered as “admixed”. The placement of genotypes on groups (or subgroups) was determined using CLUMPP v.1.1 [73], which evaluates the similarity of outcomes between population structure runs. CLUMPP output was used directly as input for Distruct v1.1 [74] in order to graphically display the results.

To validate the genetic structure revealed by the Bayesian model-based clustering two complementary approaches using the Darwin software package v6.0.10 [75] were considered: i) an unweighted neighbor-joining tree constructed based on dissimilarities between the unique genotypes (using a Simple Matching coefficient), and ii) a multivariate Principal Coordinate Analysis (PCoA).

### Genetic differentiation

Population differentiation was estimated by analyses of molecular variance (AMOVA) through Genodive [76] under two scenarios: i) three broad European geographic regions of origin of the material (North + East, West and South); and ii) the major groups (and subgroups) defined by Structure. Pairwise F_ST_ estimates for the different partitioning levels considered in each case were also obtained using Genodive [76]. Descriptive statistics were calculated for the material clustered according to geographical regions of origin as well as for each group (or subgroup) identified by the Bayesian model-based clustering method, including *Ho* and *He*, number of total alleles, number of private alleles, i.e., those only found in one (sub)division level, and number of unique alleles, i.e., those only detected in one unique accession. The software FSTAT v.2.9.3.2 [77] was applied to compute the allelic richness after scaling down to the smallest partitioning level in the different scenarios considered.

### Parentage reconstruction and relatedness between the accessions

On the basis of the SSR profiles of the unique genotypes, accessions were analyzed to infer possible parent-offspring relationships using Cervus v.3.0 software [53]. In order to reveal only robust parentages, we limited the study to the inferences of ‘two-parents offspring’ relationships and did not consider inferences of ‘one-parent offspring’ relationships where the lacking parent offers more flexibility but more speculative assignments as well, especially with only 16 SSR markers. Two criteria were considered to establish strict parentage relationships: i) a confidence level of the LOD score and ii) the Delta LOD value (defined as the difference in LOD scores between the first and second most likely two-candidate parents inferred) both higher than 95 %. Finally, an additional constraint was added to strengthen the results by limiting the maximum number of tolerated locus mismatches to only one in any inferred two-parents offspring trio, assuming that such a slight difference may be attributable to possible scoring errors, occurrence of null alleles or occasional mutational events [54, 78].

## Abbreviations

ECPGR, European cooperative programme for plant genetic resources; F_ST_, fixation index ‘F-statistics’; LOD, logarithm of odds ratio; PCR, polymerase Chain reaction; SSR, simple sequence repeat.

## Additional files

Additional file 1: List of the 2446 accessions considered in the present study with their accession code (AcceNumber), name (AcceName), the name of the providing collection (Collection), their duplicate code according to the SSR profile (FBUNQ, see text), their ploidy level (Ploidy) determined according to the occurrences of three alleles per locus (see text), their status (Analyzed) as analyzed or not-analyzed within the duplicate group (when adequate), their documented European geographic region of origin (Eur_reg_orig), their putative country of origin (Country_orig), their group assignment (Group) inferred by the Structure analysis with the highest proportion of ancestry (*qI*max), and their subgroup assignment (Subgroup) inferred by the nested Structure analysis with the highest proportion of ancestry (*qI*max nested). (XLSX 394 kb) Additional file 2: Genetic composition of cultivars clustered by country of origin for the eight subgroups inferred with Structure. For the detailed country list, see Additional file 1. The pies represent the proportion of each subgroup in each country: color codes are as per Fig. 1 a2. (TIF 5267 kb) Additional file 3: Characteristics of the 16 SSR markers used in this study with indication of the corresponding multiplex and dye. Footnotes: ^a^ [61]; ^b^ [60]; ^c^ [59]; ^d^ [62]; ^e^ Primer concentration within a given multiplex has been adjusted to get more homogeneous SSR marker amplification intensities. (XLSX 10 kb)
