# Supplementary figures and images for: Analysis of the genetic diversity and structure across a wide range of germplasm reveals prominent gene flow in apple at the European level
Source: BMC Plant Biol. 2016 Jun 8;16:130. doi: 10.1186/s12870-016-0818-0 (PMC4898379; doi:10.1186/s12870-016-0818-0)

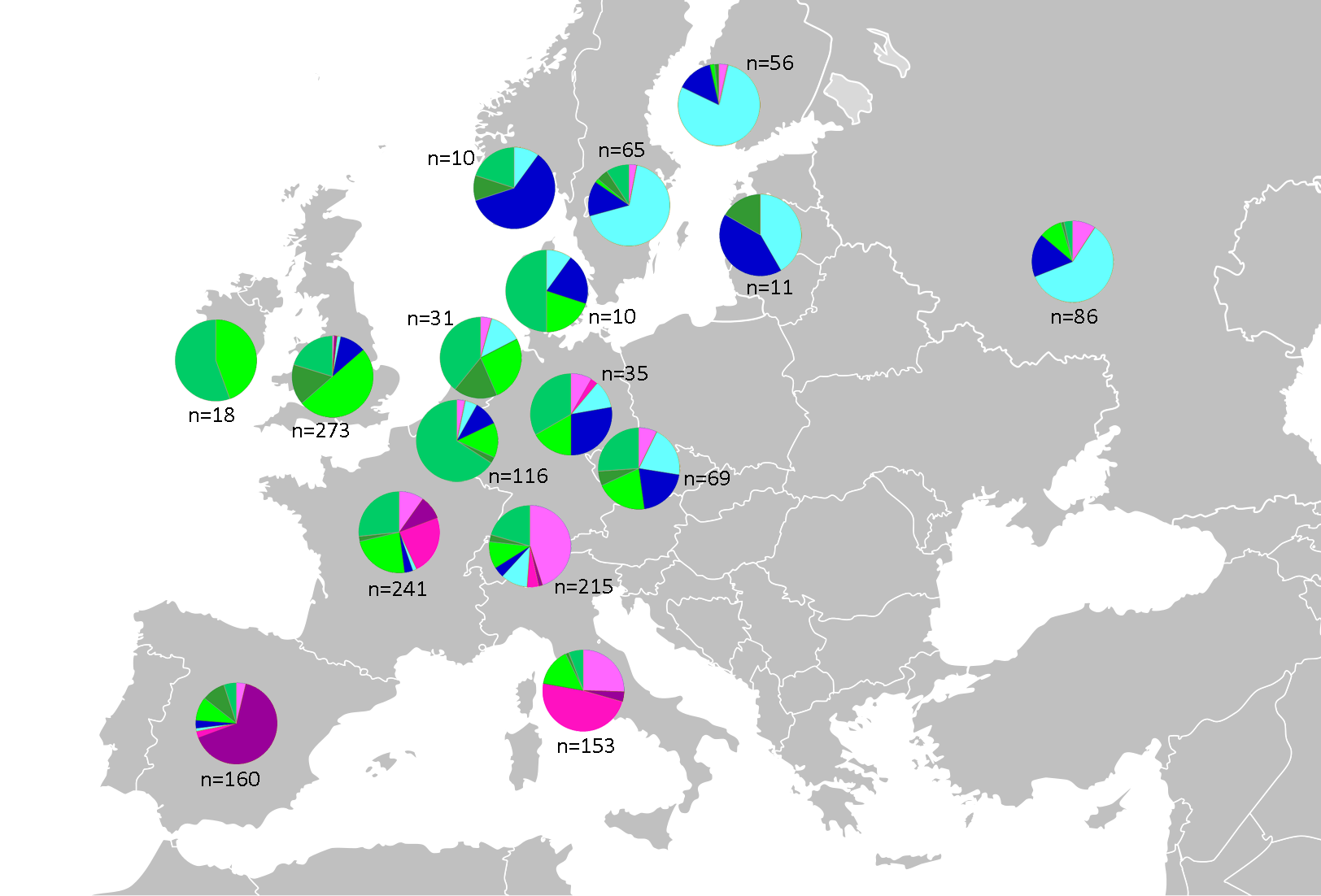

Supplement: Additional file 2: — Genetic composition of cultivars clustered by country of origin for the eight subgroups inferred with Structure. For the detailed country list, see Additional file 1. The pies represent the proportion of each subgroup in each country: color codes are as per Fig. 1 a2. (TIF 5267 kb) [file 12870_2016_818_MOESM2_ESM.tif]
